# Multiwall carbon nanotube-supported molybdenum catalysts for ammonia decomposition reaction under microwave effect

**DOI:** 10.3906/kim-1907-4

**Published:** 2020-04-01

**Authors:** Melih GÜLER, Dilek VARIŞLI

**Affiliations:** 1 Department of Chemical Engineering, Gazi University, Ankara Turkey

**Keywords:** COx-free hydrogen, ammonia, multiwall carbon nanotube, molybdenum

## Abstract

In this study, microwave-assisted ammonia decomposition reaction was investigated over molybdenum incorporated catalysts. Due to the selective, volumetric, and noncontact heating properties of the microwave system, higher conversion values could be achieved at relatively lower reaction temperatures, which is important for on-site COx-free hydrogen production. Multiwall carbon nanotube-supported molybdenum catalysts were prepared following the impregnation procedure with different metal loading (3.5%–12.5% wt%), and inductively coupled plasma, nitrogen physisorption, X-ray diffraction, and transmission electron microscopic techniques were employed to characterize the fresh and used samples. Reaction experiments were performed under the flow of pure ammonia with a gas hourly space velocity of 36,000 mL/g_cat._h for both the microwave and conventionally heated reaction systems. It was found that ammonia conversion was obtained even at 400 °C, reaching 40%, and total conversion was observed even at 450 °C, while the activities of these catalysts were negligible at a reaction temperature lower than 550 °C, in the conventional heated system, which included an electrically heated furnace. Crystals of α-Mo_2_C as well as MoO_2_ were observed in the structures of the synthesized catalysts and the formation of nitride species was more easily observable under microwave heating, possibly due to the nitridation of molybdenum carbide species during the reaction.

## 1. Introduction

Nowadays, alternative environmentally friendly energy sources have gained great attention due to the problems of using fossil fuels, such as global warming. It is known that fossil fuels, such as coal, crude oil, and natural gas, are the main resources of the world’s energy demand. However, great technological development and increased population have increased the necessity for new energy sources, since the amount of fossil fuel necessary for the near future may not be satisfactory due to the depletion of reserves.

It is known that among new energy sources, hydrogen has become attractive due to its superior properties, such as having high specific energy on a mass basis. [1–3]. Hydrogen can be produced via different routes, such as steam reforming of ethanol or dry reforming of methane. Proton exchange membrane (PEM) fuel cells are the most common way of using hydrogen to produce energy, but the purity of feed, i.e. hydrogen, should be very high to prevent poisoning of the fuel cell. However, COx components, which are produced as by-products during the synthesis of hydrogen from carbonaceous sources, may decrease the performance of the fuel cell, causing poisoning [1–3]. On-site generation of hydrogen, via the decomposition of ammonia, could help to solve the high purity, storage, and transportation problems of hydrogen, especially for mobile systems [1,2]. Ammonia is present in gaseous form at ambient temperatures and pressures, and it can easily be liquefied at 298 K under a pressure of 10 bar. Relatively inexpensive vessels could be used to easily transport and store this hydrogen-rich molecule [1].

Ruthenium incorporated catalysts have shown great activity for ammonia decomposition reaction [4–6]; however, due to the high price and scarcities of ruthenium, many studies have been devoted to much cheaper transition metal catalysts, such as Fe, Co, and Ni over the past few decades [7–12]. Ammonia decomposition reaction is an equilibrium-limited reaction and over 99% conversion could be achieved at about 400 °C according to the equilibrium curve; however, generally higher temperatures, such as 650 °C and over, have been reported in the literature for attaining total conversion over catalysts prepared with cheap transition metals. Research has concentrated on improving either the catalyst structure or reactor conditions, to obtain high conversion values at relatively low reaction temperatures, especially for on-site hydrogen production required areas.

In recent years, alternative energy sources have been considered to supply necessary energy to reactor systems. Among them, microwave energy has been used for chemical reactions, especially for endothermic reactions. In conventional heating systems, such as electrically heated furnaces, heat is transferred from the source to the target by means of conduction and convection-type heat transfer mechanisms. On the contrary, in microwave systems, heat is produced by direct conversion of electromagnetic energy at which it is required [13–15]. For the same reaction, in comparison to conventional heating, higher product yields, increased product selectivity, higher stability, and a lower amount of coke formation are obtained due to the selective, volumetric, and noncontact heating properties of microwave systems [13,14,16–18]. Different kinds of reactions have been performed via microwave heating, such as the decomposition of methane [13] and steam reforming of methanol [19]. To work with a microwave heating system, it is necessary to use a dielectric material in the reactor to absorb microwave energy [16]. Some of the materials, such as those that are carbonaceous, have the ability to absorb microwaves and have been utilized not only as a catalyst/catalyst support, but also as a microwave receptor in the reaction medium [15,20]. To date, various carbonaceous materials have been utilized as support materials in the preparation of catalysts for COx-free hydrogen from ammonia, run in conventionally heated systems, such as carbon nanotubes (CNTs) [7,21], carbon fibers [22], ordered mesoporous carbon (CMK-5) [23], porous carbon [24], and raphitized carbon [5]. However, a microwave-assisted ammonia decomposition reaction is very rare; carbon sources, namely, mesoporous carbon and carbon fiber, were used in the preparation of transition metal-incorporated catalysts. Indeed, studies that investigated mesoporous carbon-supported iron-incorporated catalysts [25] and mesoporous carbon-supported molybdenum-incorporated ones [26] are the pioneer work of microwave reactor application to the decomposition of ammonia to produce COx-free hydrogen. In these studies, it was found that iron-incorporated carbon catalysts provided complete conversion of ammonia at 450 °C (at 7.7 wt% Fe), and molybdenum-incorporated carbon catalysts exhibited total conversion at 400 °C (gas hourly space velocity (GHSV) of 36,000 mL/hg_cat_) in the microwave heated system.

CNTs have unique structural, electronic, mechanical, optical, and thermal properties and as indicated previously, they have been used as support material for different metals, such as ruthenium [5] and cobalt [27], for ammonia decomposition reaction. In these studies, different carbon sources, namely mesoporous carbon and activated carbon, as well as CNTs, were used and the results revealed some relations between the physical and chemical properties of the support and the activity of the metal species.

In the present work, the activity of multiwall CNT (MWCNT)-supported molybdenum catalysts was examined in a microwave-assisted reactor system via an ammonia decomposition reaction and compared with that obtained in a conventionally heated system. It was also expected that aside from providing knowledge about the enhanced COx-free hydrogen production under the effect of microwave energy, this study would also contribute to the literature related to the formation of the carbide species that were observed in the structure of the used catalysts.

## 2. Experimental studies

### 2.1. Synthesis of the catalysts

MWCNT-supported molybdenum-incorporated catalysts were prepared following the impregnation procedure. Ammonium molybdate (H_24_Mo_7_N_6_O_24_, Sigma-Aldrich, St. Louis, MO, USA) was used as the metal precursor and metal loading was performed by adjusting the metal wt% in the synthesis solution to between 5 and 15. The synthesis solution was prepared by solving 0.5 g of commercially available MWCNTs (95% carbon, Sigma-Aldrich) in 5 mL of aqueous ethanol (vol, 20%), and then mixing with a metal precursor. This solution was stirred at 60 °C for 3 h and then dried in an oven at 80 °C for 10 h. All synthesized samples were calcined under the flow of nitrogen at 700 °C for 5 h and then named Mo@MWCNT(X), where X referred to the wt% of the metal to the mixture of metal and MWCNT, in preparation of the synthesis solution. Before the reaction studies, reduction was applied to the calcined catalysts at 600 °C for 2 h under the flow of pure hydrogen.

### 2.2. Characterization of the catalysts

Structural properties of the MWCNT-supported molybdenum-incorporated catalysts were determined by applying different techniques. In order to determine the metal loading of the synthesized catalysts, inductively coupled plasma (ICP) analyses were performed using a Perkin Elmer DRC II model ICP-OES (Waltham, MA, USA). Nitrogen physisorption analyses were carried out at 77 K via using a Quantachrome Autosorb-6B surface area and pore size analyzer (Boynton Beach, FL, USA). Before taking measurements, the samples were degassed at 200 °C for 3 h. The Brunauer, Emmett, and Teller (BET) method was used to determine the surface area of the synthesized catalysts and the Barrett, Joyner, and Halenda (BJH) method was applied to the desorption isotherm to attain the pore size distributions. A Bruker-AXS D8 Advance A 25 diffractometer (Madison, WI, USA) with a Cu K radiation source was used to perform the X-ray diffraction (XRD) analyses, which was operated at 40 mA and 40 kV to investigate the crystal structures of the catalysts. The diffractograms were recorded in the range of 5°–90°with steps of 0.02°. The Scherrer formula (D = (K*λ) / (β *cosθ) , where K = 0.89, λ = 0.154056nm) was used to determine the average crystallite sizes. The obtained diffractograms were compared against the Joint Committee of Powder Diffraction Standards (JCPDS) and International Centre for Diffraction Data (ICDD) databases. TEM analysis was performed using a JEOL JEM 2100F high-resolution transmission electron microscope (HRTEM) (Akishima, Tokyo, Japan) with a maximum acceleration voltage of 200 kV. The samples were dispersed in ethanol and deposited on a grid covered by C-film to perform the analysis. The Raman spectrum was obtained using a Renishaw Raman microprobe (Wotton-under-Edge, UK) with a laser source at 532 nm.

### 2.3. Reaction experiments

**Figure 1:**
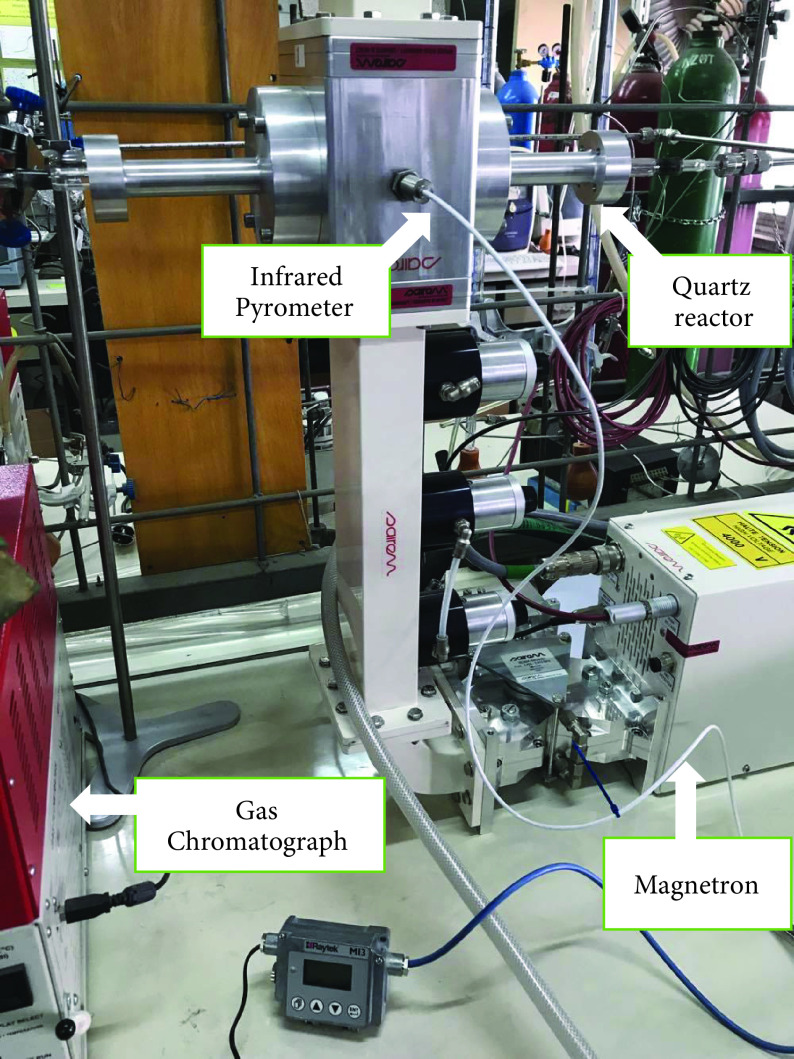
Experimental set-up: MWRS.

A microwave-heated reactor system (MWRS) was used to carry out ammonia decomposition reaction and the corresponding experimental results were compared with the data obtained from the conventionally heated reactor system (CHRS) under the same reaction conditions. In the MWRS, a Sairem microwave oven (Décines-Charpieu France) at a frequency of 2.45 GHz was used (Figure 1). Next, 0.1 g of the reduced form of the synthesized catalysts was placed into the middle of a quartz tube that was used as the catalytic bed portion in the reactor system. Quartz was selected as a material of construction for the reactor since it is transparent to microwaves [18]. During the experiment, the microwave power was sent to the catalytic bed directly and by changing the power source in the microwave system from 0.06 kW to 0.09 kW, the reaction temperature was adjusted. About 10 to 40 W of the power sent to the system was absorbed by the catalyst, which made a change in the temperature, and the cooling water absorbed the rest of microwave. A Raytek M13 infrared pyrometer (Santa Cruz, CA, USA) was used to measure the temperature, which was placed at a location looking directly into the catalytic bed. Measurements at the specified temperatures were performed after reaching steady state, and they were done from the effluent stream and the obtained data were used to calculate conversion of the ammonia (X).

In the CHRS, the experiments were repeated under the same reaction conditions, i.e. the same amount of catalyst was placed in a quartz tube and the ammonia feed flow rate was kept at 60 mL/min. Contrarily, in the MWRS, a fixed bed reactor was used in a tubular electrical furnace.

## 3. Results and discussion

**Figure 2:**
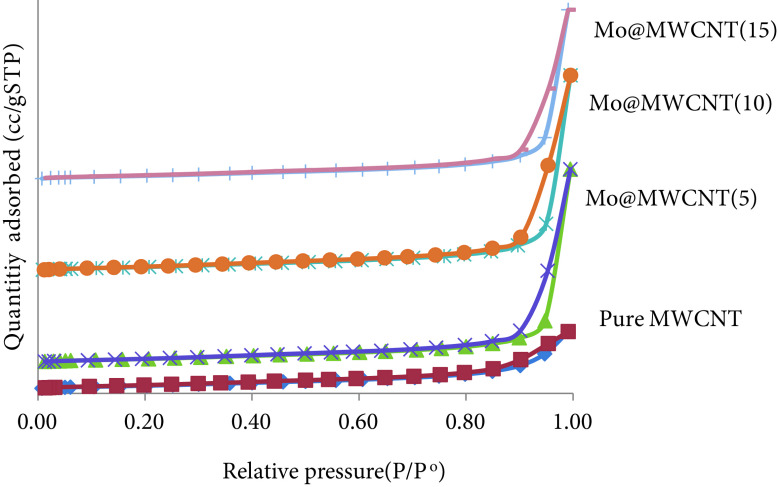
Nitrogen adsorption-desorption isotherms of the metal-incorporated MWCNT.

MWCNT-supported molybdenum-incorporated catalysts were prepared with molybdenum loading in the range of 5–15 wt%, and the results of the ICP-OES analysis, which were 3.5–12.5 wt%, showed that the impregnation procedure was achieved with some loss of the metal precursor in the synthesis solution. Nitrogen adsorptiondesorption analyses of the synthesized catalysts are presented in Figure 2, where the isotherms observed in this plot were classified as type IV according to the IUPAC classification, which indicated a mesoporous structure. The MWWCNTs had a surface area of 248 m^2^/g in pure form, and metal loading resulted in a decrease in the CNT structure, such that a catalyst with 5% metal loading (Mo@MWCNT(5)) had a surface area of 227 m^2^/g, while the one with 15% metal loading (Mo@MWCNT(15)) had a surface area of 187 m^2^/g (Table 1).

**Table 1:** Physical properties of the MWCNT-supported molybdenum catalysts.

Catalysts	Mo wt% in the synthesis solution	Mo wt% according to the ICP-OES results	Multipoint BET surface area (m^2^/g)	BJH desorption pore volume (cm^3^/g)	BJH desorption pore diameter (nm)
Pure MWCNT	-	-	247.7	0.7832	2.69
Mo@MWCNT(5)	5	3.5	226.5	2.353	31.90
Mo@MWCNT(10)	10	6.6	229.8	2.381	32.38
Mo@MWCNT(15)	15	12.5	187.1	0.3451	2.736

**Figure 3:**
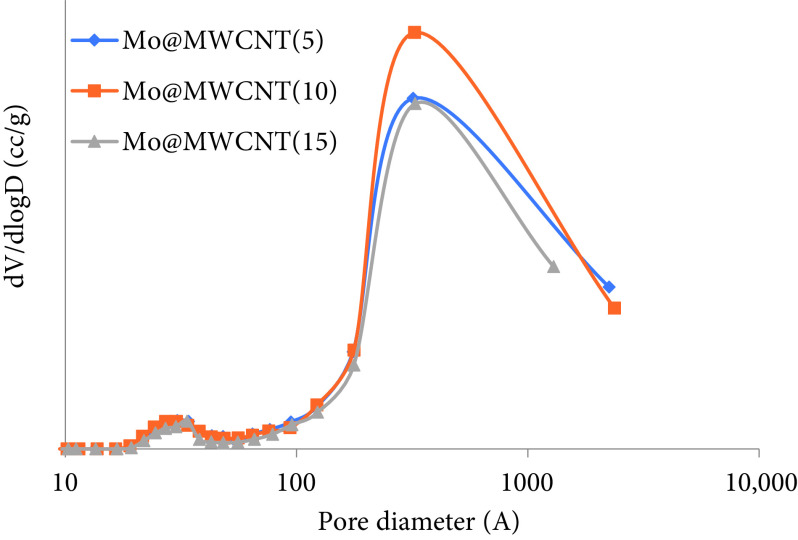
Pore size distribution of the MWCNT-supported molybdenum-incorporated catalysts.

In the pore size distribution of the synthesized catalysts, the formation of 2 peaks was observed, one was narrow with low intensity at around 2–5 nm and the other was broad with high intensity at about 10 to 100 nm (Figure 3). Similar results were revealed by Liao and Ko, who prepared mesoporous-structured Mo@MWCNT catalysts [28]. They indicated that small mesopores might be located on the inner side of the CNT and larger macropores were observed due to the space between the walls in the carbon nanostructures.

**Figure 4:**
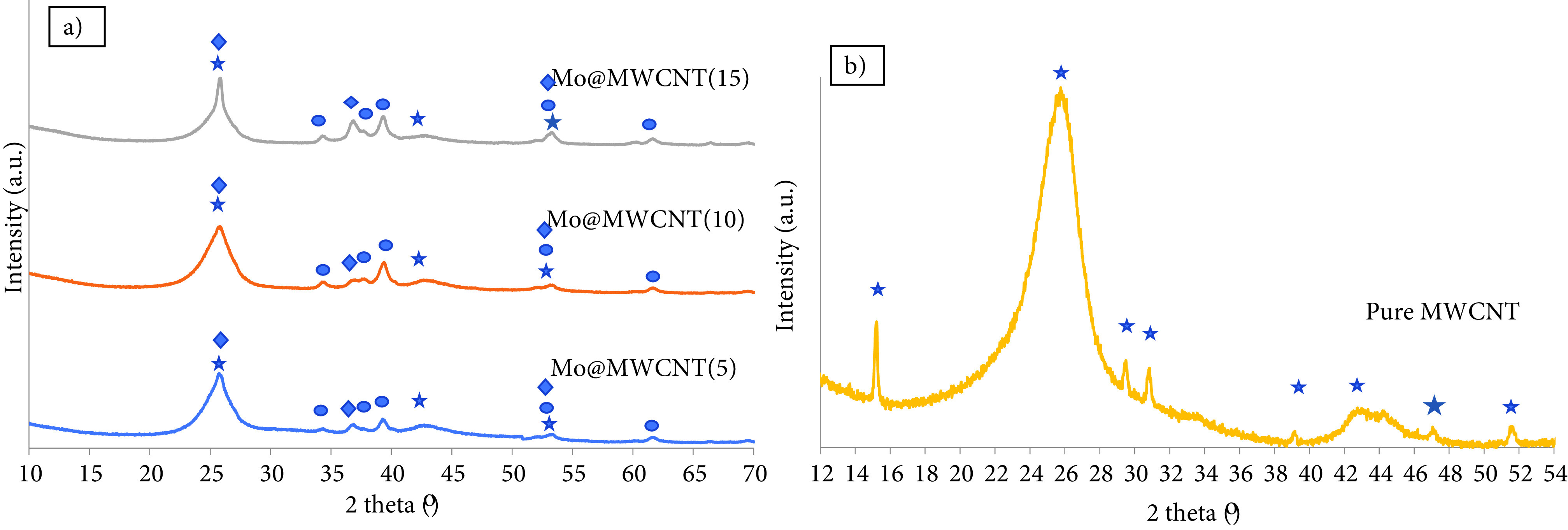
XRD analysis of the a) MWCNT-supported molybdenum-incorporated catalysts in calcined form (⋆: Carbon, ♦: MoO_2_ , •: α-Mo_2_ C) and b) pure MWCNT.

**Figure 5:**
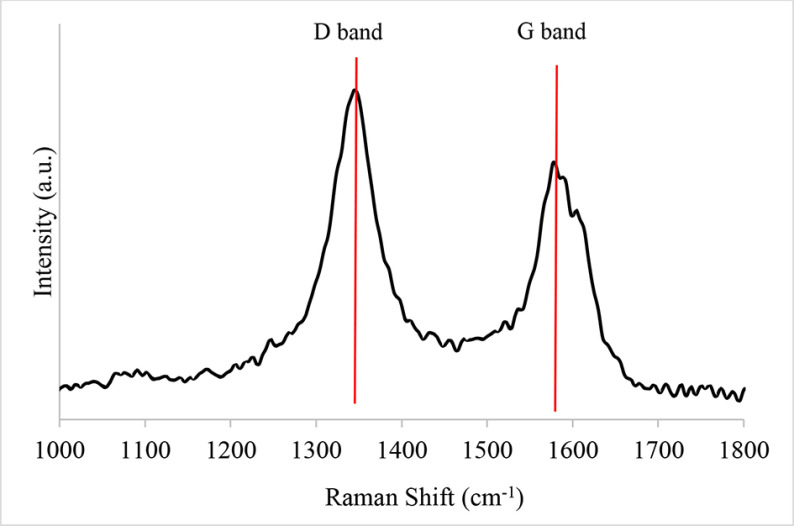
Raman spectrum of a MWCNT-supported molybdenum-incorporated catalyst.

**Figure 6:**
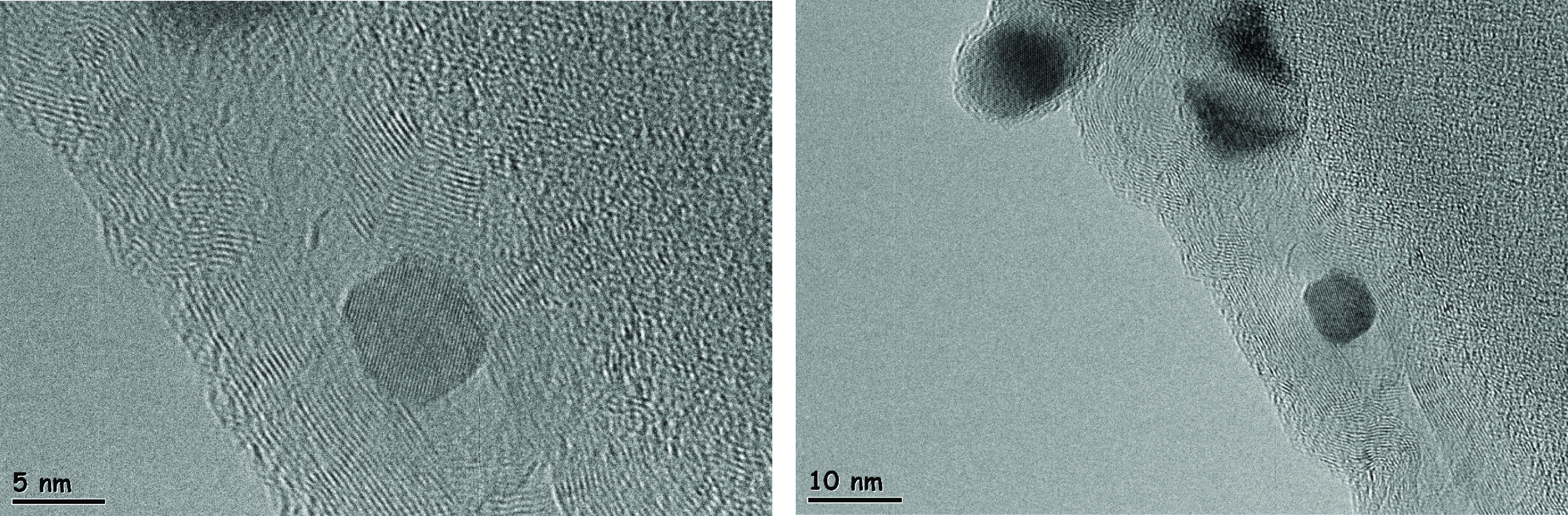
HRTEM images of the MWCNT-supported molybdenum-incorporated catalyst.

XRD analysis of the MWCNT-supported molybdenum-incorporated catalysts after being calcined, presented in Figure 4a, showed peaks at 2 theta values of 25.9°, 42.9°, 53.2°, and 78.72°corresponding to the structure of carbon (JCPDS-00-058-1638). XRD analysis was also applied to the pure form of the MWCNT and the result is presented in Figure 4b. The main peaks, located at 25.9°and 42.9°, were clearly seen in this plot and also another peak, located at 15.18°was recognized. The incorporation of metal with different loading ratios had no effect on the structure of the carbon support, except the peak located at 2 theta of 15.18°, which was hardly seen in the XRD results of the metal-incorporated ones. The Raman spectrum of the synthesized catalyst, which is presented in Figure 5, had 2 Raman-active bands at about 1580 and 1340 cm^-1^ . The peak, which was observed at 1579 cm^-1^ , was a characteristic feature of ordered graphite carbon. Another peak, which was observed at 1344 cm^-1^ , was the D band and it was attributed to the defects and disorders of the structure in carbonaceous material. Li et al. presented the Raman spectrum of different carbon materials, such as graphitic carbon, CNTs, carbon black, and so on, and they indicated a graphitic nature with a very high intensity sharp G band for at 1582 cm^-1^ and D band at 1342.9 cm^-1^ , and a shoulder D´band at 1615 cm^-1^ for commercial graphite [29]. It is known that the intensity ratio of D and G bands (*I_D_/I_G_*) is the indication of orderlinesss of the graphite structure and the corresponding value for the multiwall carbon-supported molybdenum catalyst synthesized in this study was 1.28. Li et al. mentioned that a decrease in the graphitization degree of carbon support with an increase in the *I_D_/I_G_* ratio, and they reported 1.2 for commercial graphite, which supported our result. Application of the thermal treatment resulted in the formation of MoO_2_ crystals, which were recognized with the peaks at 2 theta of 25.97°, 36.9°, 37.32°, 53.50°, and 66°(JCPDS-01-074-4517). The size of the MoO_2_ crystals was calculated as 2.89, 3.28, and 8.23 nm for the Mo@MWCNT(5), Mo@MWCNT(10), and Mo@MWCNT(15) catalysts, respectively. Molybdenum species of about 5-nm were recognized in the HRTEM images of the MWCNT-supported molybdenum catalyst (with 10 wt% loading), which is presented in Figure 6. Apart from the carbon crystals and molybdenum oxide species, molybdenum carbide was seen in the structure of the synthesized catalysts. High-intensity peaks observed at 2 theta values of 34.64°, 37.80°, 39.51°, 52.50°, 61.90°, and 69.0°all belonged to α-Mo_2_ C crystals, according to JCPDS-00-031-0871. The crystallite size of the molybdenum carbide crystals varied between 1.72 and 13.87 nm, depending on the metal loading.

Metal carbides have superior physical and chemical properties compared to metal oxides and nitrides. For the transition metal carbide, bondings were formed between the metal and carbon atoms. Their properties may have been different than their parent forms. Transition metal carbides, such as molybdenum carbides, have attracted the attention of researchers since they show similar electronic and catalytic behaviors to those of noble metals [30–36]. Different routes have been used to prepare molybdenum carbides. One of which is the temperature-programmed reduction method, which enables reaction between molybdenum oxides and a mixture of hydrogen- and carbon-containing gases, such as methane and toluene, noting that the former is used for reduction of deposited carbon and the latter is necessary as a carbon source [3,32]. Carbon sources in solid form, such as MWCNTs [3,31,33] and in liquid form, such as urea [3,35], have been also used in the preparation of carbide species. Chemical vapor deposition, the microwave-assisted method, arc melting, and mechanical activation are some examples for other methods for the preparation of molybdenum carbide and it was found that properties of carbide species, such as crystal structure, particle size, morphology, and surface area were affected by the synthesis procedure as well as carbon source [3,37]. Yang et al. reported a route that included a reaction between anhydrous molybdenum chloride with graphite or CNTs in metallic Na medium at 500 °C to synthesize nanocrystalline Mo_2_ C [31]. Ultrafine molybdenum carbide nanoparticles supported on nitrogendoped carbon nanosheets were prepared using melamine and ammonium molybdate by Xia et al. [36]. This mixed form of precursors was turned into a grafitic carbon nitride-supported MoO_x_ species at 500 °C and this intermediate phase was converted into black powder with a further increase in heat, indicating the formation of carbon and Mo_x_C at 800 °C. Theerthagiri et al. performed carburization of hydrothermally synthesized α-MoO_3_ nanowire with urea, which was used as a carbon source and thermal treatment was performed at 700 °C under a nitrogen atmosphere [35]. In the work of Vitale et al., the effects of temperature, synthesis time, C/Mo ratio, and the presence/lack of hydrogen on the formation of molybdenum carbide materials with different phases were investigated in detail. Ammonium heptamolybdate and sucrose were used, and hexagonal and cubic phases of the Mo_2_ C were obtained, depending on the conditions [34]. In the present study, ammonium heptamolybdate and MWCNTs were used as precursors, and Mo_2_ C was obtained after thermal treatment at 700 °C under nitrogen flow.

**Figure 7:**
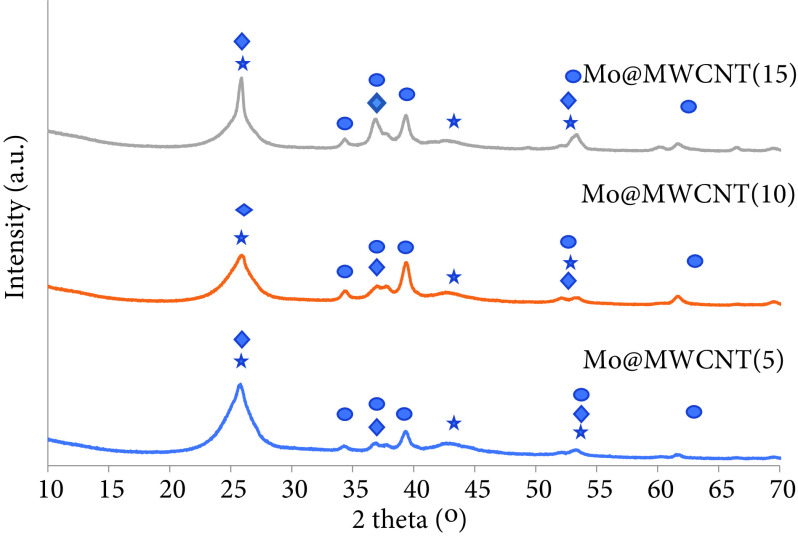
XRD analysis of the MWCNT-supported molybdenum-incorporated catalysts in reduced form (⋆: Carbon, ♦: MoO_2_ , •: α-Mo_2_ C).

XRD results of the catalysts after being reduced, presented in Figure 7, revealed the presence of α-Mo_2_ C crystals at sizes of 13.48, 14.01, and 14.05 nm for Mo@MWCNT(5), Mo@MWCNT(10), and Mo@MWCNT(15), respectively. Oxide species were still present after reduction step and the MoO_2_ crystal sizes were 2.91, 3.87, and 10.83 nm for the synthesized catalysts. Metallic molybdenum was barely observed. As seen in Figures 4 and 7, molybdenum carbide and molybdenum oxide species were present before and after the reduction, indicating that either reduction may have been insufficient to obtain a pure metallic phase with high intensity or the formed pure metallic molybdenum phase may have been easily oxidized before the measurement was taken.

**Figure 8:**
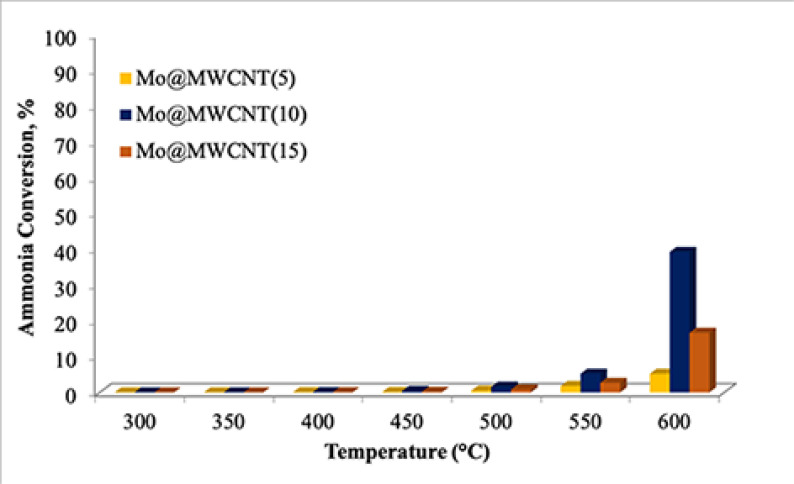
Ammonia conversion values obtained from the CHRS (GHSV_*NH3*_: 36,000 mL/g_cat_ .h).

Activities of the MWCNT-supported molybdenum-incorporated catalysts were negligible at a reaction temperature lower than 550 °C, when they were used in the CHRS (GHSV_*NH3*_: 36,000mL/g_cat_ .h), as presented in Figure 8. Even though an increase in the temperature favored the reaction, conversion of ammonia was still low, i.e. at about 40%. The optimum metal loading that showed the highest activity was 10%.

**Figure 9:**
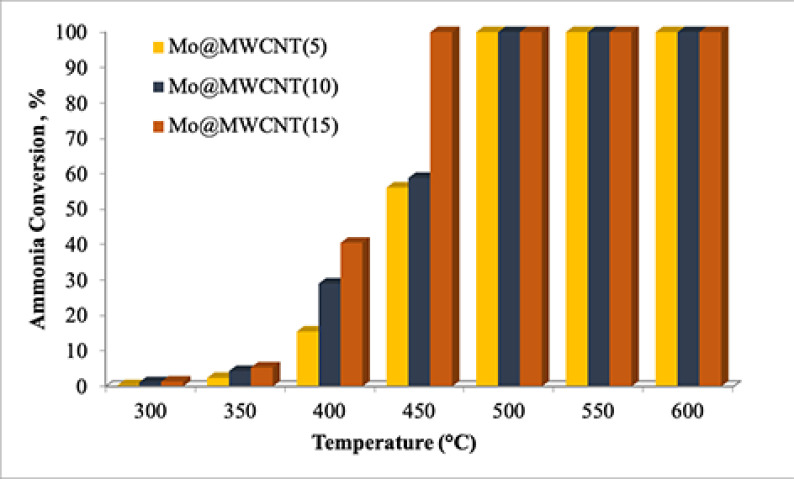
Activities of the catalysts in the MWRS (GHSV_*NH3*_: 36,000 mL/g_cat_ .h).

MWCNT-supported molybdenum-incorporated catalysts showed better activity when MWRS was used, under similar reaction conditions, i.e. the GHSV of pure ammonia was 36,000 mL/hg_cat_ (Figure 9). Ammonia conversion was realized even at temperatures of 400 °C, reaching 40%. Any increase in the reaction temperature favored the activity, as was expected. Synthesized catalysts, with 5% and 10% molybdenum, namely Mo@MWCNT(5) and Mo@MWCNT(10), showed 60% ammonia conversion and the one prepared at the highest metal loading achieved total conversion. Time on stream data for the catalyst under microwave radiation was investigated by testing the synthesized catalyst, such as Mo@MWCNT(10), at 500 °C for 300 min and no decrease in the activity of the catalyst was observed.

The CHRS and MWRS are different from each other in terms of heating mechanisms and this can explain the variation of the activity of the MWCNT-supported molybdenum catalysts from one system to another. In the conventional system, an electrical furnace was used in order to supply the necessary heat to the reactor and transfer of heat took place from the electrical resistance to the walls of furnace and the cavity of the furnace, first, and then through the sample via sequential conduction and convection mechanisms, and this might have caused a decrease in the temperature in a radial way. Durka et al. revealed that the temperature very close to the wall would be higher than the core temperature due to these heat transfer mechanisms [38]. On the contrary, microwave energy is transferred from the source to the active sites of the catalyst, so heat is generated in the catalytic bed. Therefore, the center part of the catalytic bed is supposed to have the highest temperature [38–39], and this situation is defined as volumetric heating and an inverse temperature profile is formed under this condition [38]. A higher core temperature in comparison to the wall temperature (i.e. inverse temperature profile), in addition to the prevention of heat loss during heat transfer, would be possible reasons for attaining better activity in MHRS [16,14].

It is known that the differences observed in the microwave absorption ability of metaloxide crystals and that of the support materials may cause the formation of hot spots, which are also identified as microplasmas [38]. This might be considered as another reason to explain the higher activity obtained in microwave systems. Having higher local (microscale) temperatures of the hot spots than the average bulk temperature, may result in an increase conversion [18,40,38].

In the literature, the microwave-specific effect discussed by Stiegman et al. was also given as a reason for attaining higher activity in microwave-focused reactions [41,42]. The Boudouard reaction was run under microwave irradiation in comparison to conventional convective heating and lower apparent activation energy and lower enthalpy of reaction were reported under microwave irradiation [41]. They discussed that the reduction observed in the apparent enthalpy of the reaction under microwave irradiation could have been due to the additional energy being put into the carbon by means of microwaves. A specific microwave effect on the mechanism was proposed as the reason for a great change in the apparent thermodynamic of the reaction, resulting in higher conversion values at lower reaction temperatures. They suggested a plausible mechanism where the formation of electron-hole pairs on the surface of the carbon under constant radiation could be considered as the radical anions and cations, respectively, behaving like active sites, and that their interaction with CO2 enhanced the activity. Moreover, polar groups that would be present on the oxidized surface, could couple with radiation and their selective interfacial heating might increase desorption of product CO from the surface. Specific microwave effects could be also considered to explain better activity of the MWCNT-supported molybdenum catalysts in the ammonia decomposition reaction, but there is no direct proof of that at present. It is expected that further studies using transition metal-incorporated carbon catalysts may provide new insight to clarify the specific microwave effects on this reaction.

**Figure 10:**
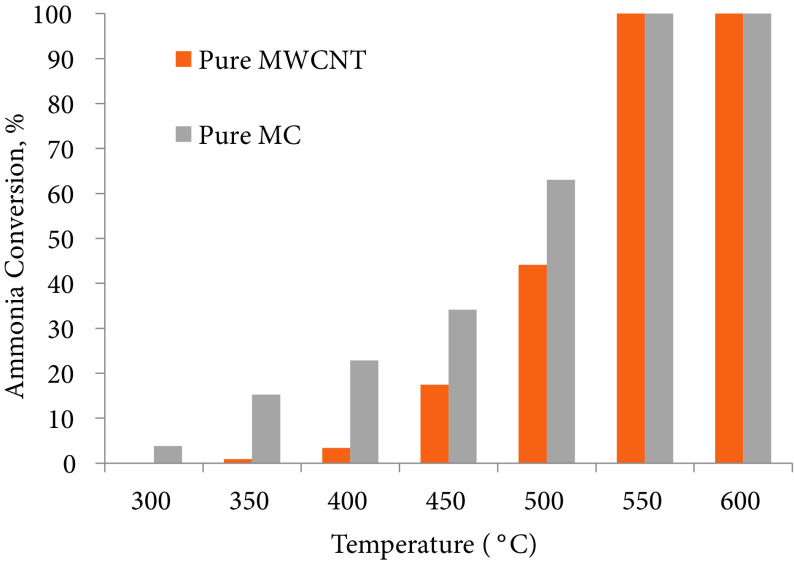
Activities of the support materials in pure form in the MWRS (GHSV_*NH3*_: 36,000 mL/g_cat_ .h).

Carbon based materials enable heating of reaction medium due to their good microwave absorbing properties. Moreover, they provide better distribution of metal species due to their high surface area and porous structure. The catalytic activity of pure support materials on the decomposition reaction of ammonia was also examined. Moreover, the activity of pure mesoporous carbon in the ammonia decomposition reaction was negligible, even at the highest reaction temperature when a CHRS was used [25]. In the present work, a similar result was found for the MWCNT material. To evaluate the effect of microwave energy on the catalytic activity of the support material, both of the supports, namely MWCNT and mesoporous carbon, were run in a MHRS, following the same experimental conditions that were applied for the metal-incorporated carbon-based catalysts, and the results are presented in Figure 10. Interestingly, both of the carbonaceous materials enabled conversion of ammonia under microwave heating, even though they did not show any activity under conventional heating. As seen in Figure 10, MWCNT, in its unsupported form, exhibited 44% ammonia conversion at 500 °C and total conversion was achieved at 550 °C under microwave effects. The MWCNTs used in these experiments were commercially obtained and their purity was 95%, and the mesoporous carbon used in the experiments had purity of 99.95%.

Previously, Zhang et al. indicated the high activity of commercially obtained CNTs containing a residual amount of Co or Fe nanoparticles for NH_3_ decomposition [7]. In their work, commercially obtained Cocontaining carbon nanotubes (4.1 wt% Co) exhibited 100 ammonia conversion at 700 °C (SV: 20,000 h^-1^) and the Fe-containing carbon nanotubes (2.8 wt% Fe) exhibited 75.1% ammonia conversion at the same temperature with a space velocity of 5000 h^-1^ . The chemical vapor deposition method over metal-based solid catalysts, such as Fe, Co, Ni, and Mo, has been used to produce CNTs, and transition metals resulting from the production process could be present in a highly dispersed state, which remain in the raw material and are supposed to be very active in the reactions [7]. In this study, MWCNTs were used at reaction temperatures less than 600 °C and the ammonia flow was set to 36,000 mL/g_cat_ .h., with no activity observed in the CHRS. According to the results presented in Figure 10, the MWCNTs had good activity in the MHRS, and this could be explained by either the catalytic activity of the residual amount of metals (Mo, Fe), which would probably be present in the support structure, under the effect of the microwaves, or graphitization of the carbon support itself, under the effect of microwaves. It was really difficult to observe any crystals belonging to the metals in the XRD analysis of the pure MWCNTs, presented in Figure 4b, meaning that they would have been dispersed in the structure. Meanwhile, the enhanced activity of mesoporous carbon, which was used in almost pure form, would highly support the latter suggestion. It is known that a high graphitization degree facilitates the nitrogen desorption step, which is the limiting step, in the ammonia decomposition reaction [27].

**Figure 11:**
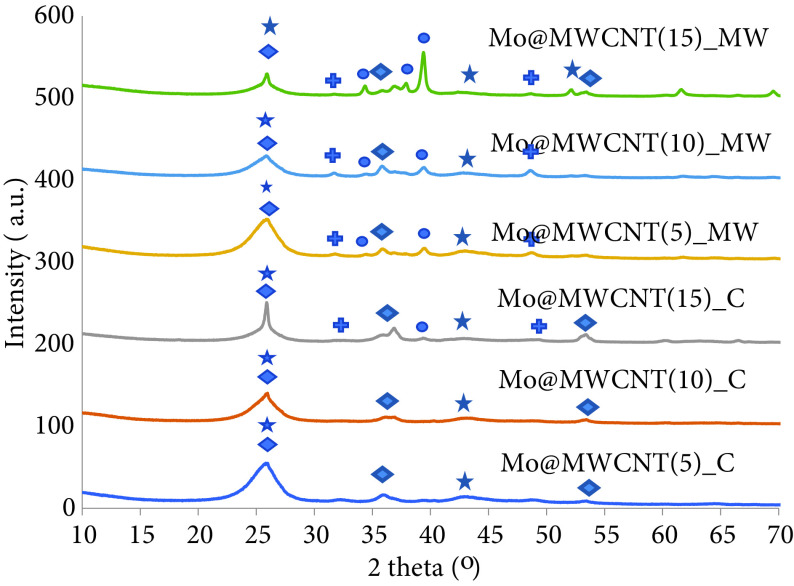
XRD analysis of the MWCNT-supported molybdenum-incorporated catalysts after use (⋆: Carbon, ♦: MoO_2_ , •: α-Mo_2_ C, +: Mo5 N6) .

In order to see the effects of microwaves on the structure of the catalyst, XRD analyses were applied to the used samples. The samples, which are shown as Mo@MWCNT(X)_MW in Figure 11, refer to the catalysts that were used in the MHRS, and samples given as Mo@MWCNT(X)_C in the same figure refer to samples run in the CHRS. When the ammonia decomposition reaction was performed under conventional heating, oxide forms were observed in the catalyst structures. Molybdenum carbide and molybdenum nitride crystals, with very low intensities compared to the oxide phase, were recognized. When the catalysts were run under microwave heating, oxide, nitride, and carbide species were seen in the XRD analysis of the catalysts. Peaks observed at 2 theta of 32.32°, 36.7°, and 49.7° belonged to Mo5 N6 , according to JCPDS-00-051-1326. While the crystals of molybdenum carbide became larger in the case of the highest metal-loaded catalyst Mo@MWCNT(15), this was most probably due to the effect of microwaves in the formation of the carbide species, while that of molybdenum nitride became smaller for the same catalysts. The size of the α-Mo_2_ C crystals were 6.33, 8.32, and 22.75 nm, for Mo@MWCNT(5), Mo@MWCNT(10), and Mo@MWCNT(15), respectively.

Even though the formation of molybdenum carbide was seen in the calcined form of the catalysts, it was much more clearly seen in the structure of the used catalysts that had been taken from the MHRS in comparison to the samples taken from the CHRS. It is known that the application of microwaves to synthesize molybdenum carbide provides some advantages, such as a very short synthesis time, comparatively low synthesis temperature, and it occurs in a 1-step process [43,44]. Vallance et al. synthesized molybdenum carbide using carbon and molybdenum metal or trioxide in 90 s using a microwave oven running at 800 W [43]. Yacob et al. applied microwave irradiation (800 W) to the mixture of an ammonium heptamolybdate solution and carbon black powder. They reported that molybdenum carbide was formed after 2 min and molybdenum oxide converted to molybdenum, so Mo_2_ C was formed [44].

Several research groups have worked on the formation of molybdenum nitride crystals from molybdenum oxides during the decomposition of ammonia [45–47]. A detail in situ XRD analysis was carried out by Tagliazucca et al. during reaction on a MoO_3_ catalyst, which was prepared following an impregnation procedure with a molybdic acid solution and activated carbon [46]. They indicated that MoO_3_ nanopaticles had been converted to molybdenum bronze (HxMoO_3_) and MoO_2_ particles at 350 °C, and then hexagonal molybdenum nitrides were formed between 450°and 500 °C, under ammonia flow (15,000 mL/g_sample_ h). For complete nitridation, high temperatures (>650 °C) are required. Synthesis of MoN phase with a reaction between Mo films with ammonia was also achieved at 998 K for 2 h [48]. In the present study, the nitridation process was not applied to the MWCNT-supported molybdenum-incorporated catalysts before the ammonia decomposition reaction; however, X-ray analysis of the used catalysts revealed molybdenum nitride compounds, which would be explained by the reaction conditions having enhanced the formation these compounds.

According to the theoretical calculations, adsorbed N atoms tend to form surface or subsurface nitride and further bulk nitride after the cleavage of N-H bond. Thus, nitridation of Mo_2_ C might occur from surface to bulk [49]. Moreover, there is a possibility of oxidation of the molybdenum nitride crystallites that result in the formation of molybdenum oxide, since metal nitride species are air sensitive [50]. Nitride species with a very low intensity observed in the structure of the synthesized catalysts could have been formed due to the nitridation of oxide or carbide species.

In the literature, there are some studies related with the application of molybdenum incorporated catalysts to ammonia decomposition reaction, for example, Ji et al. [51] studied Co-Mo bimetallic catalysts at a reaction temperature interval between 350°and 600 °C (GHSV of 36,000 mL/g_cat_ h) and Liang et al. [45] worked on nitrided MoNx/Al_2_O_3_ and NiMoNx/Al_2_O_3_ , and ammonia conversion of 99% was reported at 650 °C (GHSV of 1800 h^-1^) by Liang et al. [45]. Podila et al. [50] indicated total conversion of ammonia on molybdenum nitride catalysts, which were used after the nitridation process, at 600 °C with a GHSV of 6000 h^-1^ . Moreover, some examples of the application of different metals, such as Co, Fe, and Ru, with different support materials, such as CNT and SiO2 , are summarized in Table 2. Most of these studies were performed in a CHRS [7,21,24,40,46,49,50,52–55]. Even though activity of the MWCNT-supported molybdenum catalysts were lower than that given in the literature, the MHRS greatly enhanced the activity of these catalysts, even when compared with the activity of ruthenium-based catalysts. Studies based on microwave-assisted ammonia decomposition reaction revealed that carbon material supported metal-incorporated catalysts, showing greatly enhanced activities under microwave radiation [25–26,56]. There are 2 main contributions of these carbon materials to better activities, one of which is being a good metal support. They did not collapse during metal loading and they generally provided a high surface area. The other is due to their good microwave absorption properties, which enable them to heat up the reaction medium within a few seconds. The latter may be pointed out as being more important than the former.

**Table 2:** Catalytic activities of different catalysts for the ammonia decomposition reaction.

						
MoN@C	25.9	600	15,000	90	Conventional	24
Mo_2_C	Bulk	600	36,000	71	Conventional	49
MoO_3_-G	-	600	15,000	80	Conventional	46
MoN	Bulk	600	6000	97.2	Conventional	50
MoNx/Al_2_O_3_		650	1800	99	Conventional	40
La-CoMoNx/CNT	10	600	11,000	97.63	Conventional	21
Co/MWCNT		500	6000	74.6	Conventional	52
Co-containing CNT		700	20,000	~100	Conventional	7
Fe-containing CNT		700	5000	75.1	Conventional	7
Ru/La2O3		450	18,000	72.8	Conventional	53
Ru/CNT		450	18,000	69.5	Conventional	54
Ru-containing SiO_2_		400	6000	84	Conventional	55
Co@MWCNT		600	36,000	40	Conventional	This study
Co@MWCNT		450	36,000	~100	Microwave	This study

## 4. Conclusions

In this study, MWCNT-supported molybdenum catalysts with different metal loading (3.5–12.5 wt%) and a surface area in the range of 187–227 m^2^/g were prepared and used for ammonia decomposition reaction in 2 different systems, namely MHRS and CHRS. α-Mo_2_ C, as well as MoO_2_ , were the crystals observed in their structures after being thermally treated under nitrogen flow. Activities of the MWCNT-supported molybdenumincorporated catalysts were negligible at a reaction temperature lower than 550 °C, when they were run in the CHRS (GHSV_*NH3*_: 36,000 mL/g_cat_ .h). On the other hand, they showed better activity when the MWRS was used, under the same reaction conditions. Ammonia conversion was realized even at temperatures of 400 °C, reaching 40%, and an increase in the reaction temperature favored the activity, i.e. total conversion was observed even at 450 °C. Even though the supports (MWCNTs) in pure form were not active in the ammonia conversion when the CHRS was used, with the effect of microwaves, it showed total conversion over 550 °C. Formation of nitride species were much more observable under microwave heating compared to conventional heating, possibly due to the nitridation of molybdenum carbide species during the reaction.

There are 2 main contributions of these carbon materials to better activities, one of which is being a good metal support. They did not collapse during metal loading and they generally provided a high surface area. The other is due to their good microwave absorption properties, which enable them to heat up the reaction medium within a few seconds.

Microwave energy has superior properties when compared to conventionally heated systems, such as being a selective and volumetric heating method; thus, enhancing the activity of the synthesized catalysts during the ammonia decomposition reaction. Moreover, specific microwave effects on the reaction are supposed to occur and further studies using transition metal-incorporated carbon catalysts may provide new insights toward clarifying these effects.
